# Hypoxia Marker Carbonic Anhydrase IX Is Present in Abdominal Aortic Aneurysm Tissue and Plasma

**DOI:** 10.3390/ijms23020879

**Published:** 2022-01-14

**Authors:** Katarina Grossmannova, Monika Barathova, Petra Belvoncikova, Viliam Lauko, Lucia Csaderova, Jan Tomka, Tomas Dulka, Jaromir Pastorek, Juraj Madaric

**Affiliations:** 1 Department of Cancer Biology, Institute of Virology, Biomedical Research Center, Slovak Academy of Sciences, Dúbravská Cesta 9, 84505 Bratislava, Slovakia; katarina.laposova@savba.sk (K.G.); petra.belvoncikova@savba.sk (P.B.); lucia.csaderova@savba.sk (L.C.); 2 Department of Laboratory Medicine, National Institute of Cardiovascular Disease, Pod Krásnou Hôrkou 1, 83101 Bratislava, Slovakia; viliam.lauko@nusch.sk; 3 Department of Vascular Surgery, National Institute of Cardiovascular Disease, Pod Krásnou Hôrkou 1, 83101 Bratislava, Slovakia; jan.tomka@nusch.sk (J.T.); tomas.dulka@nusch.sk (T.D.); 4 MABPRO, a.s., Dúbravská Cesta 2, 84104 Bratislava, Slovakia; jaromir.pastorek@gmail.com; 5 Department of Angiology, National Institute of Cardiovascular Disease, Pod Krásnou Hôrkou 1, 83101 Bratislava, Slovakia; juraj.madaric@nusch.sk

**Keywords:** abdominal aortic aneurysm, carbonic anhydrase IX, hypoxia-inducible factor 1, hypoxia

## Abstract

Abdominal aortic aneurysms (AAA) are a significant cause of premature deaths worldwide. Since there is no specific treatment for reducing AAA progression, it is crucial to understand the pathogenesis leading to aneurysm wall weakening/remodeling and identify new proteins involved in this process which could subsequently serve as novel therapeutic targets. In this study, we analyzed the presence of the hypoxia-related proteins carbonic anhydrase IX (CA IX), hypoxia-inducible factor 1α (HIF-1α), and AKT as the key molecule in the phosphoinositide-3-kinase pathway in the AAA wall. Additionally, we used a blood-based assay to examine soluble CA IX (s-CA IX) levels in the plasma of AAA patients. Using western blotting, we detected CA IX protein in 12 out of 15 AAA tissue samples. Immunohistochemistry staining proved CA IX expression in the media of the aneurysmal wall. Evaluation of phosphorylated (p-AKT) and total AKT showed elevated levels of both forms in AAA compared to normal aorta. Using ELISA, we determined the concentration of s-CA IX >20 pg/mL in 13 out of 15 AAA patients. Results obtained from in silico analysis of *CA9* and aneurysm-associated genes suggest a role for CA IX in aneurysmal wall remodeling. Our results prove the presence of hypoxia-related CA IX in AAA tissues and indicate a possible role of CA IX in hypoxia-associated cardiovascular diseases.

## 1. Introduction

Abdominal aortic aneurysm is defined as an enlargement of the infrarenal or suprarenal aorta to a diameter of at least 3 cm. AAA affects about 3% of the population aged over 50, with four times higher incidence in males compared to females. Aneurysms are mostly asymptomatic, but weakening of the arterial wall can lead to its rupture and fatal bleeding into the retroperitoneal cavity [1]. Although many of the processes leading to AAA development still remain unclear, recent studies have shown that key pathophysiological features of AAA include chronic inflammation [2], extracellular matrix degradation [3], vascular smooth muscle cell (VSMCs) phenotype modulation, VSMCs apoptosis [4], and hypoxia [5].

Hypoxia is broadly studied as a biologically and clinically important phenomenon, especially in cancer as well as in the pathogenesis of various cardio-vascular diseases [5]. In the wall of large arteries, luminal blood diffusion is the main source of nutrients and oxygen for the cells located on the luminal side, while perfusion via vasa vasorum provides nourishment to the cells located on the abluminal side. Any alteration in these processes, or a change in oxygen consumption by cells, creates a hypoxic microenvironment [6]. At the molecular level, the adaptation of cells to hypoxic stress is regulated by hypoxia-inducible factors (HIFs), which mediate the expression of over 100 genes involved in important signaling pathways [7].

In aneurysms, hypoxia and hypoxia-inducible factor 1 (HIF-1) stimulate inflammatory processes by the infiltration of macrophages, T cells and B cells, proinflammatory factor secretion, and upregulation of matrix metalloproteinase (MMP) secretion by smooth muscle cells [8,9,10]. Imbalance of MMPs and tissue inhibitors of MMPs (TIMPs) destroys the structural integrity of the aortic wall through elastin and collagen degradation [11]. Moreover, recent studies indicate that hypoxia stimulates the proliferation and migration of VSMCs, thus causing the active remodeling of a vessel wall [12,13].

Carbonic anhydrase IX is a unique protein highly overexpressed in hypoxic cells/tissues. It is a transmembrane zinc metalloenzyme, which plays a crucial role in intracellular pH maintenance [14]. Moreover, CA IX activity stimulates tumor cell migration and invasiveness [15]. The key regulator of CA IX expression is transcription factor HIF-1 [16]. Soluble CA IX in serum or plasma can be used as a diagnostic marker in patients with renal cell carcinoma [17], breast cancer [18], non-small cell lung cancer [19], rectal cancer [20], or testicular cancer [21]. However, data about the role of serum/plasma CA IX concentration in patients with non-cancerous diseases are missing.

In the present study we tested the presence of hypoxia-related proteins CA IX and HIF-1α in AAA tissues by western blot and analyzed the localization of CA IX in the media of the AAA wall by immunohistochemistry. Moreover, by determination of plasma CA IX values using an enzyme-linked immunosorbent assay (ELISA) we evaluated the concentration of soluble CA IX (s-CA IX) in plasma samples from AAA patients suggesting the presence of s-CA IX in hypoxia-related cardiovascular diseases.

## 2. Results

### 2.1. Patient and AAA Characteristics

Baseline characteristics of patients are shown in Table 1.

Table 2 shows chronic medication of patients before surgery.

Based on preoperative diagnostic imaging (CT angiography plus ultrasound), in 9 out of 15 patients the aortic aneurysm was characterized by an intraluminal thrombus formation with thickened aortic wall.

### 2.2. Expression of CA IX in Human AAA Tissues

**Figure 1:**
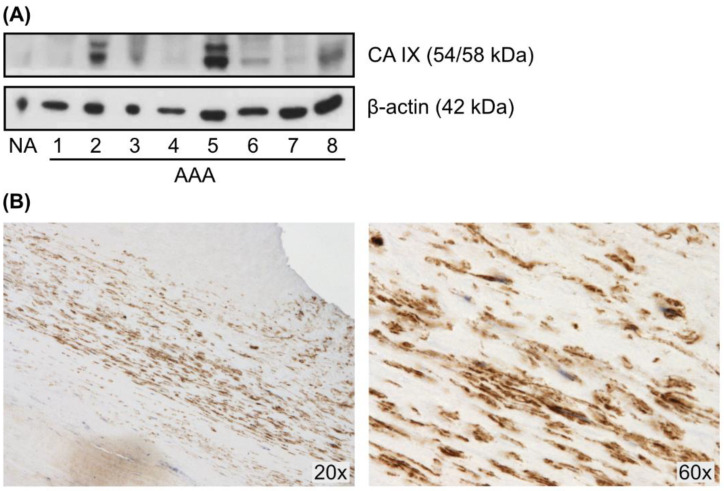
Representative western blot of CA IX protein expression in human AAA tissues. (**A**) CA IX level was determined by western blot analysis of proteins extracted from human abdominal aortic aneurysms (AAA) and a pool of normal aortas (NA). AAA samples showing the twin band representing CA IX protein were marked as positive. Detection of β-actin served as a loading control. (**B**) Representative images (objective magnification 20× and 60×) of CA IX staining in AAA wall by immunohistochemistry.

Using western blotting, we determined the expression of CA IX in 15 AAAs. As a negative control we used a commercially available protein lysate from a pool of normal aortas. CA IX expression was confirmed in 12 out of 15 AAA tissue samples (80%). Five out of 12 positive samples showed a high level of CA IX protein (representative western blot is presented in Figure 1A). On the contrary, a pool of control aortas demonstrated the absence of a CA IX signal (Figure 1A). CA IX levels were not influenced by age, gender, or cigarette smoking status, and did not correlate with aneurysmal diameter. The mean diameter of all CA IX positive aneurysms was 61 ± 13 mm, the mean diameter of five aneurysms with the highest level of CA IX was 56 ± 4 mm, while the mean diameter of three CA IX negative aneurysms was 72.7 ± 6.7 mm.

Importantly, we proved the presence of CA IX in the AAA wall also by immunohistochemistry. CA IX-positive staining was localized in the media of aneurysmal wall specimens (Figure 1B). CA IX staining was absent in tissues proved negative by western blotting (data not shown).

### 2.3. Expression of HIF-1α, p-AKT, and AKT in Human AAA Tissues

**Figure 2:**
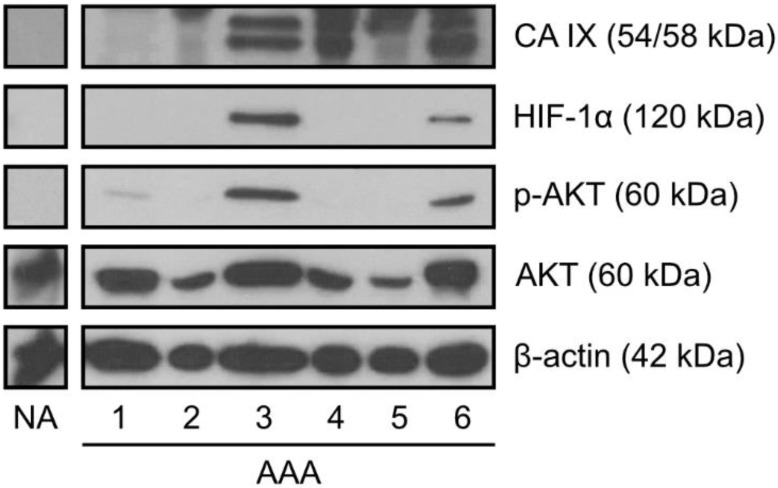
Representative western blots of HIF-1α, p-AKT, and AKT expression in human AAA tissues. Protein levels were determined by western blot analysis of proteins extracted from human abdominal aortic aneurysms (AAA) and a pool of normal aortas (NA). Detection of β-actin served as a loading control.

Since CA IX expression is triggered in hypoxic conditions via HIF-1-mediated transcriptional activation [16], we examined the expression of the oxygen-regulated α subunit of HIF-1 transcription factor in AAA tissues. Western blot data showed the presence of HIF-1α in three AAA protein lysates, all of which also demonstrated a high expression of CA IX (representative western blot is presented in Figure 2). HIF-1α was absent in the pool of control aortas.

AKT is a key molecule in the phosphoinositide-3-kinase (PI3K) pathway involved in the regulation of multiple cellular processes. Moreover, AKT has been shown to play an important role in AAA formation [22]. To evaluate the status of AKT phosphorylation, we analyzed human AAA tissues and a pool of control aortas for the presence of phosphorylated Ser473 of AKT protein (p-AKT). Although a level of phosphorylation varied between samples, we detected p-AKT in 11 out of 15 AAAs. p-AKT was absent in normal aorta lysate. In contrast, a high level of total AKT was detected in all samples (AAAs and control aorta lysate) with only moderate differences in protein levels (Figure 2). However, we found no correlation between CA IX abundance and AKT phosphorylation on Ser473.

### 2.4. Quantification of Soluble CA IX in Plasma Samples from AAA Patients

Plasma/serum level of s-CA IX can serve as a biomarker for cancer diagnostics, especially for renal cell carcinoma [17]. Soluble CA IX can be detected as a result of shedding from the hypoxic cell surface mediated by disintegrin and metalloproteinase ADAM17 [23]. Since we found the expression of CA IX protein in AAA tissues, we performed ELISA, detecting CA IX to evaluate the presence of s-CA IX in the plasma samples obtained from the same AAA patients. We determined an s-CA IX concentration >20 pg/mL (ranging from 22.8 to 513.5 pg/mL, with an average of 105.8 pg/mL) in 13 out of 15 AAA patients. In two patients positive for plasma CA IX we did not detect any CA IX protein in AAA tissue, indicating that the presence of s-CA IX can be associated with an aneurysm-unrelated disease. In one of the patients there was a significant stenosis of the common femoral artery; the second patient suffered from significant renal stenosis and carotid artery stenosis. Soluble CA IX concentration detected in control plasma samples obtained from 15 healthy individuals ranged from 2.934 to 28.096 pg/mL, with an average of 9.553 pg/mL. Our results show a significant difference (*, *p* < 0.05) between soluble CA IX levels detected in the plasma of AAA patients and control healthy individuals.

### 2.5. Summary of the Obtained CA IX and HIF-1α Results and Detailed Information about Individual Patients

In Table 3 we summarized detailed characteristics about each AAA patient with our results: *CA9* mRNA expression analyzed by semiquantitative PCR (Section 2.6), CA IX and HIF-1α detection by western blot, and s-CA IX concentration determined by ELISA.

### 2.6. In Silico Analysis and Expression Profiling of Selected Proteins

To evaluate a possible CA IX function in AAA formation, we performed an in silico analysis of abdominal aortic aneurysms to correlate *CA9* gene with genes related to AAA formation. Using four different datasets, we analyzed the correlation between *CA9* and genes involved in matrix remodeling/degradation (*COL1A1*, *COL3A1*, *MMP8*, *MMP9*, *TIMP1*, *TIMP2*, *TIMP3*, *CTSB*, *CTSD*, *CTSK*, *CTSL1*, *CTSL2*, *CTSS*), altered smooth muscle cell phenotype/proliferation (*ACTA2*, *SPP1*, *MYOCD*, *FOXO4*, *SRF*), and inflammation/neovascularization (*VEGFA*).

The results (Table 4) showed a positive correlation between *CA9* and *ACTA2, COL1A1, CTSL1, MYOCD*, and *TIMP3* in three databases and *VEGFA* in all four databases. The positive correlation of *CA9* and *COL3A1*, *SPP1*, *TIMP1*, and *TIMP2* was proved in two databases. The correlations between *CA9* and *CTSL1* were positive in three out of the four databases, and the correlation between *CA9* and *CTSB*, *CTSD* and *CTSK* was positive only in one database.

**Figure 3:**
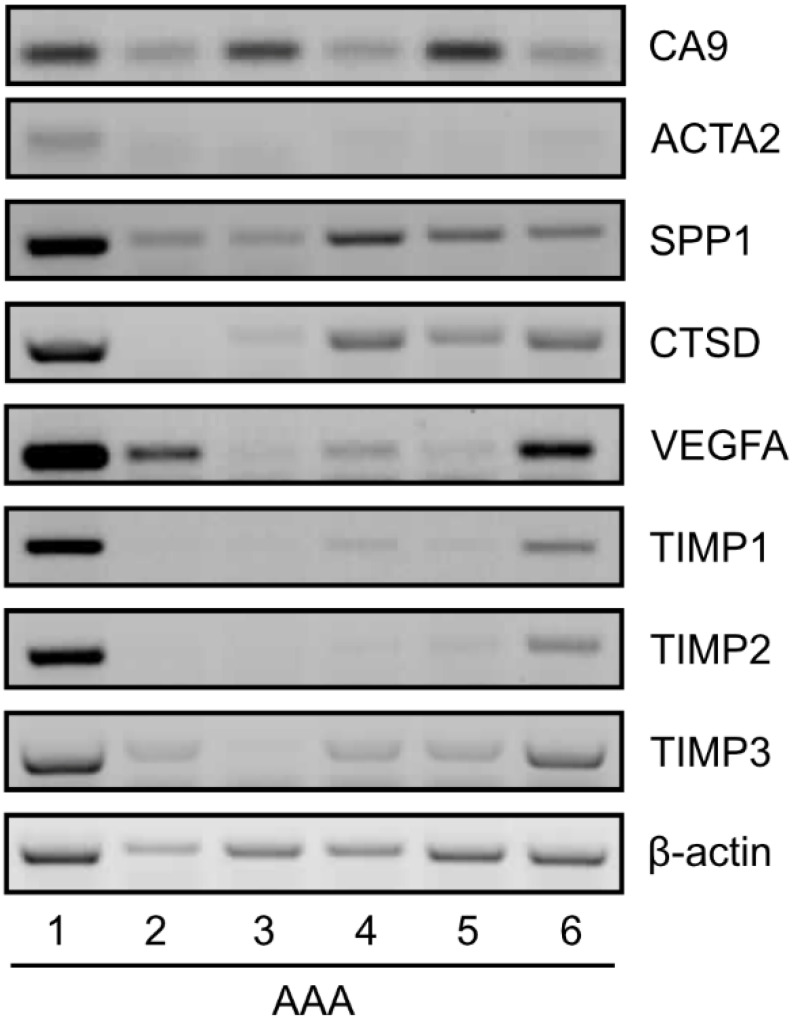
Representative semiquantitative PCR of *CA9*, *ACTA2*, *SPP1*, *CTSD*, *VEGFA*, *TIMP1*, *TIMP2*, and *TIMP3* in human AAA tissues. Detection of β-actin served as a control.

To examine the gene expression of selected genes in our AAA tissue samples, we isolated total RNA, reverse transcribed it to cDNA, and performed semiquantitative PCR. The results showed that *CA9* gene was expressed in all AAA tissues. Moreover, we detected mRNA of other genes analyzed in silico and showed that 45% were positive for *ACTA2*, 100% were positive for *SPP1*, 82% were positive for *CTSD*, 91% were positive for *VEGFA*, 73% were positive for *TIMP1* and *TIMP2*, and 82% were positive for *TIMP3* (Figure 3).

## 3. Discussion

Carbonic anhydrase IX has become the protein of interest because it represents the most accurate marker of hypoxia. It is induced in hypoxic areas via the HIF-1-mediated pathway, allowing cells to survive in conditions lacking an oxygen supply [16]. Hypoxia can also occur as a result of diseases of the vascular wall leading to a disruption of blood circulation. Such hypoxia may additionally contribute to the formation of many pathologies of the vessel wall, including atherosclerosis, pulmonary hypertension, chronic venous diseases, or arterial aneurysms [5].

Abdominal aortic aneurysm disease is a life-threatening, chronic degenerative condition, predominantly of the infrarenal segment of the abdominal aorta. A growing body of evidence links hypoxia and HIF-1α expression/activity to AAA development/rupture. Intraluminal thrombus, a common feature of AAA, attenuates oxygen flow to the AAA wall, leading to cellular hypoxia and wall weakening [24]. Hypoxia may stimulate inflammatory cell infiltration and increased matrix metalloproteinase activity, resulting in elastin destruction and aneurysmal dilatation [25].

Taken together, hypoxia in AAA suggests the presence of CA IX in abdominal aneurysms. To date, there has only been one study monitoring CA IX in aortic aneurysm tissues. Confirmed by histology, CA IX-positive staining was found in adventitia in 20 out of 30 ascending aortic aneurysm patients’ tissues and it was significantly associated with increased ascending aortic dilatation, macrophages and B cell infiltration, and inflammation [26].

In the present study, we analyzed the presence of CA IX in cases of abdominal aortic aneurysm and demonstrated the expression of CA IX protein in 12 out of 15 AAA tissues using western blotting. On the contrary, a CA IX signal was absent in control aortas. Five out of 12 CA IX-positive samples showed a high level of this protein. Variations in CA IX amount are consistent with results of the Wang group who reported large differences in HIF-1α mRNA expression detected within a human aneurysm cohort [9].

To determine whether CA IX correlates with aortic dilatation, we analyzed the AAA diameters at the time of surgery. The mean diameter of highly positive aneurysms from our study was 56 ± 4 mm, while the mean diameter of three CA IX-negative aneurysms was 72.7 ± 6.7 mm. This is in contrast with Niinimaki’s study, where the mean diameter of the ascending aorta was significantly increased in CA IX-positive samples as compared to negative samples (63 ± 3 vs. 53 ± 2 mm) [26]. Similar to our data, Wang et al. showed elevated mRNA levels of HIF-1α and its target genes (suggesting the presence of hypoxia) in aneurysm tissues, but mRNA levels did not correlate with aneurysmal diameter [9]. In order to determine the relationship between CA IX and aneurysm diameter more accurately, it is necessary to examine more AAA samples.

To prove the existence of hypoxic areas in AAA tissues, we analyzed the level of the most widely used marker of hypoxia—HIF-1α. Interestingly, we were able to detect HIF-1α only in 3 out of 5 strongly CA IX-positive AAA samples. The reason we detected HIF-1α protein in such a small number of samples (despite CA IX positivity) could be due to the instability and fast degradation of HIF-1α. Moreover, CA IX has a much longer half-life in reoxygenated cells (about 38 h) [27] than HIF-1α (about 5–10 min) [28]. Due to its high stability, CA IX is detectable not only in hypoxic tissues but also in post-hypoxic/reoxygenated tissues. Taken together, these data highlight CA IX as a suitable hypoxia marker, since analysis of HIF-1α can provide false negative results.

We also confirmed the presence of CA IX protein in AAA by immunohistochemistry. CA IX-positive staining was found in the media of an aneurysmal specimen, suggesting its presence in vascular smooth muscle cells. The localization of hypoxic areas in aneurysmal aorta differs in prior studies. Similar to our study, Billaud et al. revealed the evidence of chronic hypoxia in the media of aneurysmal ascending aortic specimens using immuno-based detection of the glucose transporter *GLUT1*, a gene target of HIF-1. Interestingly, in aneurysmal adventitia, mRNA levels of *HIF-1α* and the HIF-1 downstream gene targets vascular endothelial growth factor (*VEGF*) and metallothionein (*Mt-1A*) were down-regulated [29]. In contrast, Niinimaki et al. showed positive CA IX staining in the adventitia (at the vicinity of the media) of a dilated ascending aorta wall [26], and Wang et al. detected nuclear HIF-1α immunostaining in adventitia of human and mice abdominal aortic aneurysm tissues [9]. In a recent study by Tanaka et al., HIF-1α immunoreactivity was positive in all layers of the AAA wall (intima, media, and adventitia), especially in macrophages and smooth muscle cells [30].

Numerous studies have shown that CA IX has a crucial role in cancer progression via its enzyme activity and/or non-catalytic mechanisms [15]. The presence of CA IX in the aneurysmal wall raises the question of what role CA IX could play in AAA development. To answer this question, we performed in silico analysis to find out which genes in AAA correlate with CA IX expression. AAAs are characterized by decreased vascular elasticity, but according to some analyses this is caused not only due to aortic wall degeneration, leading to a passive lumen dilatation, but also by its active and dynamic remodeling, in which vascular smooth muscle cells play a central role [31]. In recent studies, it was shown that VSMCs can undergo a process known as a phenotype switch—differentiation of VMSCs from a contractile into a synthetic phenotype characterized by impaired contractility but also by a stronger ability of proliferation, migration, and production of extracellular matrix components—and that HIF-1α belongs to the factors affecting this event [32,33]. In our in silico analysis, we investigated the correlation coefficient of *CA9* with α-SMA (*ACTA2*, smooth muscle α-actin, biomarker of contractile VMSC) and with the expression of osteopontin (*SPP1*, OPN, biomarker of synthetic VSMC). The results showed that both *ACTA2* and *SPP1* are positively correlated with *CA9*. This is in contrast with Liu et al.’s study which demonstrated HIF-1-dependent decreased expression of α-SMA and increased expression of osteopontin [33]. To verify the results in silico, we performed semiquantitative PCR and evaluated expression of *ACTA2* and *SPP1* in AAA tissues from our study. We found that 45% of *CA9*-positive AAA tissues were expressing *ACTA2* and 100% of tissues were expressing *SPP1*. The presence of OPN in CA IX positive tissues is in line with investigations of tumor tissues which showed a positive correlation between *CA9* and *SPP1* mRNA expression in different types of cancer [34,35]. In silico analysis also showed a positive correlation between *CA9* and *MYOCD* (myocardin)—a cardiac muscle- and smooth muscle-specific transcriptional coactivator of serum response factor (SRF), activating a subset of SRF-dependent genes and encoding contractile and cytoskeletal elements. Thus, myocardin has been identified as a master regulator of smooth muscle cell differentiation/phenotypic switching. A link between hypoxia and myocardin was shown in human VSMC where hypoxia increased myocardin at both mRNA and protein levels [36]. Moreover, Zhu et al. reported the myocardin-regulated endothelial–mesenchymal (EndMT) transition of vessel endothelial cells into smooth muscle-like cells induced by hypoxia [37]. Mounting evidence indicates that endothelia and EndMT are also involved in adult cardiovascular diseases. It is also known that CA IX drives the epithelial–mesenchymal transition in cancer cells, a process very similar to EndMT.

The initiation and progression of AAA depends also on extracellular matrix (ECM) remodeling, characterized by the destruction of the major components of the arterial wall ECM—elastin and collagen. This destruction is caused mainly by cysteine protease cathepsins and matrix metalloproteinases. In our in silico analysis, we proved a positive correlation between *CA9* and *MMP8* in two out of four databases. The data respecting the correlation between *CA9* and different types of cathepsins (*CTSB*, *CTSD*, *CTSK*, *CTSL1*, *CTSL2*, *CTSS*) were inconsistent, so we focused on the expression of *CTSD* in AAA tissues and revealed that 82% of *CA9*-positive tissues were positive for *CTSD* mRNA. It is known that pH changes in the extracellular environment and lysosomes directly affect cathepsin functions and so CA IX-mediated acidic extracellular pH activates some lysosomal cathepsins which then degrade the host’s extracellular matrix [38].

Other features contributing to remodeling of the arterial wall are inflammation and changes in microcirculation. Our results from the in silico analysis showed a positive correlation between *CA9* and *VEGFA*, a potent angiogenic and proinflammatory factor. Moreover, PCR proved a 91% match in *VEGFA* and *CA9* presence in AAA tissues. VEGFA expression was shown to be increased in AAA compared to normal aorta [39], and since both CA IX and VEGFA are induced by HIF-1α, the correlation between *CA9* and *VEGFA* is consistent with these findings.

Taken together, it is possible that CA IX plays a role in matrix remodeling/degradation and altered smooth muscle cells phenotype/proliferation—an active remodeling of the vascular wall, but to prove that further experiments are needed.

CA IX has a crucial role in acid–base balance regulation and intracellular pH maintenance, leading to acidification of the extracellular microenvironment. This acid–base homeostasis is governed by “transport metabolon” formed by carbonic anhydrase IX and various transport proteins, such as bicarbonate transporters AE1 and NBcE1 [40], or sodium/calcium exchange member 1 (NCX1), regulating intracellular calcium levels and thus affecting VSMC elasticity by their calcification [41,42]. Extracellular acidic pH also facilitates the proteolytic activity of many proteases and, in addition, results in E-cadherin degradation and impairment of cell–cell contacts [43]. CA IX is functionally involved in this process by reducing E-cadherin-mediated adhesion of cells via interaction with β-catenin [44]. Although this phenomenon is associated especially with carcinogenesis, it could possibly lead to disintegration of contacts among vascular cells present in acidic microenvironments, thus affecting aneurysmal wall integrity.

Accumulating evidence suggests an important role of the PI3K/AKT signaling pathway in VSMC phenotype regulation [33] and AAA formation [45]. Ghosh’s group found that AAA patients had a significantly higher level of AKT with phosphorylated Thr308, Ser473, and total AKT than control aortas [22]. Moreover, AKT is a crucial factor with respect to the gender differences of various cardiovascular diseases [46]. In our study, we detected an elevated level of phosphorylated Ser473 of AKT in 11 out of 15 AAA tissues in comparison with control aortas. However, unlike Ghosh’s group’s study, which showed a significant increase in the level of phosphorylated Thr308 of AKT in male compared with female AAA tissues, suggesting its role in AAA sex-specific differences [22], we did not observe any variation between phosphorylated Ser473 levels in male and female AAA tissues.

Recent reports have provided evidence that the extracellular domain of CA IX can be released into body fluids and serve as a prognostic biomarker in patients with renal cell carcinoma [17], breast cancer [18], non-small cell lung cancer [19], or rectal cancer [20]. Less studied is the possibility of plasma/serum CA IX detection in patients with non-cancerous diseases connected with reduced oxygen supply/hypoxia. A study from 2018 examining the detection of soluble CA IX in patients with cirrhosis showed significantly higher serum CA IX levels in cirrhotic patients (median s-CA IX concentration was 482 pg/mL, ranging from 11 to 1921 pg/mL) compared to a healthy cohort [47]. In the current study, we quantified the level of plasma CA IX in AAA patients. We determined s-CA IX concentration >20 pg/mL (ranging from 22.8 to 513.5 pg/mL) in 13 out of 15 AAA patients. However, in one patient positive for s-CA IX, we did not detect CA IX protein in AAA tissue, indicating that the presence of s-CA IX is associated with AAA-unrelated disease. According to anamnesis, this patient had no history of oncological comorbidity. On the contrary, not all plasma from patients with CA IX-positive AAA tissues contained s-CA IX at a concentration higher than 20 pg/mL. We can speculate that this phenomenon may be related to a different level/activity of functional ADAMs (metalloproteinases required for CA IX shedding [23]) in AAA tissues, since their activity can be inhibited by TIMPs. A recently published study exploring the association between soluble CA IX and atherosclerotic outcome measures showed that plasma CA IX was detectable in only a small number (14%) of participants and so it was not a meaningful biomarker of cardiovascular disease outcome measures in the CODAM (Cohort On Diabetes and Atherosclerosis Maastricht) cohort [48]. Despite a small number of plasma samples in our study, soluble CA IX concentration was higher than 20 pg/mL in 86.6% of them. The average s-CAIX level of AAA patients was 105.8 pg/mL, a value significantly elevated compared to the s-CA IX concentrations of healthy control plasma samples with an average of 9.553 pg/mL. To determine if s-CA IX could serve as a suitable biomarker for aneurysms, more samples should be analyzed. To date, the function of the soluble form of CA IX circulating in the bloodstream has not been elucidated. Therefore, it should be noted that s-CA IX could be not only found in body fluids of oncological patients but also in patients with other hypoxia-related diseases.

Currently, there are no reliable biomarkers of AAA presence or markers of progression of small AAAs. Better understanding of AAA pathophysiology is an important prerequisite not only for early detection and addressed surveillance of patients with small AAA [49] but also for the development of novel and more personalized treatment options. Of interest, pharmacological inhibition of ADAM17, the membrane-bound enzyme and regulator of multiple transmembrane proteins by proteolytic processing, is effective in suppressing thoracic aortic aneurysm formation, as well as its progression in mice [50].

We realize that this study has limitations in terms of number of samples. However, given the available information from some studies suggesting that the direct extrapolation from animal models to humans is not suitable for mechanisms underlying hypoxia-related vascular disease [51], we wanted to analyze human AAA tissues to see the real state of pathogenesis in humans.

Detection of CA IX in AAA patients opens new insights leading to an understanding of AAA development and, in addition, suggests a possible role of CA IX in cardiovascular diseases.

## 4. Materials and Methods

### 4.1. Study Subjects

We analyzed tissue samples of 15 consecutive AAA patients (M:F 13:2, average age 72 ± 7 years) scheduled for open aortic aneurysm repair. The mean diameter of abdominal aorta was 63 ± 13 mm. Two patients had symptoms related to dilated abdominal aorta (symptomatic AAA), other patients were classified as asymptomatic. Based on preoperative diagnostic imaging (CT angiography plus ultrasound), in 9 out of 15 patients the aortic aneurysm was characterized by intraluminal thrombus formation with thickened aortic wall.

History of cancer disease was present in three patients: one case of prostate cancer, one case of urinary bladder cancer, and one case of colorectal cancer disease. In nearly ¾ of patients (73%), the known advanced atherosclerotic process (coronary artery disease, or peripheral artery disease, or carotid stenosis) with significant arterial stenosis was present. Baseline characteristics of patients are given in Table 1; chronic medication of patients before surgery is described in Table 2.

### 4.2. Human Aortic Aneurysm Specimen Collection and Sample Preparation

With local ethics committee approval (ID: 850/17), surgical specimens of AAA tissues were obtained during therapeutic open surgeries between April 2017 and March 2018. Human tissue samples were collected and handled according to the Declaration of Helsinki. The patients provided specific informed consent for the research use of the tissue and blood specimens prior to inclusion in the study. Surgeries and plasma isolations were performed at the National Institute of Cardiovascular Diseases, Bratislava, Slovakia. Portions of AAA tissues were frozen and stored at −80 °C (for protein isolation) or immediately fixed with 10% neutral buffered formalin (for immunohistochemical staining). Protein lysate prepared from normal aortas pooled from 49 males/females, ages: 15–65; cause of death: trauma and sudden death (Human Aorta Protein Medley) was purchased from Clontech. Patient plasma samples were isolated from EDTA anticoagulated blood samples and stored at −20 °C. The plasma of 15 individuals without a cardiovascular disease diagnosis was collected with the approval of the local ethics committee (EK/BmV-03/2021).

### 4.3. Western Blotting

Proteins from specimens were extracted by homogenization in ice-cold lysis buffer (50 mM TrisHCl pH 7.4; 150 mM NaCl; 1% Triton X100; 0.05% NaDOC; 1 mM EDTA; 0.1% SDS; Protease Inhibitor Cocktail Tablets (Roche, Basel, Switzerland)) and protein concentration was determined using the bicinochoninic acid kit (Thermo Fisher Scientific, Waltham, MA, USA). Total protein extracts (50 μg/lane) were mixed with Laemmli buffer, separated in 10% SDS-PAGE, and transferred onto polyvinylidene difluoride membrane (Immobilon TM-P, Millipore, Darmstadt, Germany). After 30 min blocking with 5% non-fat dry milk in 0.1% Tween in PBS, membranes were incubated with primary antibodies: hybridoma medium containing mouse monoclonal antibody M75 recognizing PG-domain of CA IX (prepared at the Institute of Virology, Biomedical Research Center, 1:3 in blocking buffer, 1 h, RT); anti-β-actin (Cell Signaling, Danvers, MA, USA, 1:5000 in 3% BSA in TBS-T buffer, 1 h, RT); anti-HIF-1α (BD Transduction Laboratories, USA, 1:250 in 3% BSA in TBS-T buffer, O/N, 4 °C); anti-p-AKT (Ser 473) (Cell Signaling, 1:1000 in 3% BSA in TBS-T buffer, O/N, 4 °C); anti-AKT (Cell Signaling, 1:1000 in 3% BSA in TBS-T buffer, O/N, 4 °C). After washing, membranes were incubated with HRP-conjugated anti-mouse or anti-rabbit antibody (Dako, Santa Clara, CA, USA, 1:5000 in blocking buffer, 1 h, RT). Protein signals were visualized using enhanced chemiluminescence.

### 4.4. ELISA

Plasmatic CA IX concentrations were determined in triplicate with a commercially available ELISA DuoSet Human Carbonic Anhydrase IX kit (R&D Systems, Inc., Minneapolis, MN, USA) according to the manufacturer’s instructions. An unpaired *t*-test with Welch’s correction was performed in GraphPad Prism 9.3.0 to compare the measured mean plasmatic CA IX concentrations of 15 AAA samples and 15 samples from healthy individuals.

### 4.5. Immunohistochemistry

Paraffin-embedded tissues were sectioned and mounted onto glass slides. The 5 μm-thick slides were deparaffinized and rehydrated. CAIX was detected using the DAKO EnVision™ FLEX System according to the manufacturer’s instructions. Sections were incubated with anti-CAIX antibody M75 (1:25) over night at 4 °C. Negative controls were prepared by omission of the primary antibody. The stained sections were examined using a Leica DM4500B microscope and images were captured with a Leica DFC480 camera.

### 4.6. In Silico Analysis

Microarray data series from a publicly available GEO repository were reanalyzed to evaluate the expression of carbonic anhydrase IX and other selected genes and their possible correlations in human abdominal aortic aneurysm (AAA) samples. The following series were used: GSE57691 (49 AAA samples), GSE7084 (6 AAA samples), GSE47472 (14 AAA samples), and GSE98278 (48 AAA samples). Expression profiles of selected genes were acquired using the GEO2R analysis tool and possible correlations of selected genes with *CA9* were assessed in MS Excel by calculating Pearson correlation coefficients.

### 4.7. RNA Isolation and PCR

Total RNA from tissue samples was isolated using TRI Reagent solution (1 mL of TRI Reagent per 100 mg tissue), followed by reverse transcription of 1 μg RNA with the High-Capacity cDNA Reverse Transription kit (Applied Biosystems, Foster City, CA, USA) according to the manufacturer’s instructions. PCR reactions were performed using DreamTaq Green PCR Master Mix (2×) (Thermo Fisher Scientific, Waltham, MA, USA) and the primers listed in Table 5. After an initial denaturation at 95 °C for 3 min, the amplification program was set as follows: denaturation at 95 °C for 30 s, annealing at 60 °C for 30 s, and extension at 72 °C during 30 s for a total of 30–33 cycles, and finally 5 min at 72 °C. The PCR products were then analyzed on a 1% agarose gel.

## 5. Conclusions

Our results prove the presence of hypoxia-related proteins CA IX and HIF-1α in AAA tissues and elevated s-CA IX concentrations in AAA patient plasma specimens. Although the exact mechanism is not fully elucidated, our results open a new window to understand the development of AAA and to clarify the role of CA IX in diseases associated with decreased oxygen levels in the cellular microenvironment. CA IX and the mechanisms responsible for its increase could become potential targets of future therapeutic interventions in patients with AAA. Further studies focused on hypoxia and CA IX could provide novel insights into the molecular mechanisms of aortic disease with the potential for effective therapeutic strategies.

## Figures and Tables

**Table 1:** Patient characteristics.

N	15
Age (years)	72 ± 7
M:F	13:2
BMI (kg/m^2^)	26.79 ± 6
AAA diameter (mm)	63 ± 13
Arterial hypertension	13 (87%)
Diabetes mellitus	3 (20%)
Smoking	11 (73%)
HLP	6 (40%)
CAD/PAD/carotid disease	11 (73%)
EF LV (%)	59 ± 4
sCreat (µmol/L)	100 ± 34
CKD	7 (47%)
CRP (mg/L)	5.5 ± 5.3
History of oncological comorbidity	3 (20%)

AAA—abdominal aortic aneurysm, BMI—body mass index, CAD—coronary artery disease, CKD—chronic kidney disease, CRP—C-reactive protein, EF LV—ejection fraction of left ventricle, F—female, HLP—hyperlipidemia, M—male, PAD—peripheral artery disease.

**Table 2:** Medication before aortic surgery.

N	15
Aspirin/clopidogrel	10 (67%)
Statin	9 (60%)
Atorvastatin	9 (60%)
ACEI/ARB	10 (67%)
BB	10 (67%)
Ca blocker	8 (53%)
Long term anticoagulation	1 (7%)

ACEI—angiotensin-converting enzyme inhibitor, ARB—angiotensin receptor blocker, BB—beta-blocker, Ca blocker—calcium channel blocker.

**Table 3:** Summary table with detailed information about patients: *CA9* mRNA expression results, detection of CA IX and HIF-1α proteins, and level of the soluble form of CA IX (s-CA IX).

Patient Number	1.	2.	3.	4.	5.	6.	7.	8.	9.	10.	11.	12.	13.	14.	15.
**Age**	70	61	67	66	66	75	70	85	66	72	78	77	73	79	80
**Male/Female (M/F)**	M	M	F	M	M	M	M	M	M	F	M	M	M	M	M
**BMI (kg/m^2^)**	25.4	25.3	30	31	23.9	35	20.1	31.9	35.6	17	21.1	33.3	28.7	17.3	26.4
**Symptomatic AAA (0/1)**	0	0	0	0	0	0	0	0	0	1	0	0	0	1	0
**Diameter AAA (mm)**	47	44	54	60	57	58	77	65	70	72	58	76	90	72	50
**Thrombus in AAA (0/1)**	1	1	1	0	1	0	1	1	1	1	1	1	1	1	0
**Fusiform/ Saccular AAA (FF/S)**	FF	FF	FF	FF	FF	FF	FF	FF	FF	FF	FF	FF	FF	FF	S
**Multiple aneursyms (0/1)**	0	1	0	0	0	0	0	1	0	0	1	1	1	1	0
**History of malignity (0/1)**	0	0	0	1	0	0	0	0	0	1	0	0	0	1	0
**AHT (0/1)**	1	1	1	1	1	1	1	0	1	1	1	1	1	1	0
**History of smoking (0/1)**	1	1	1	1	1	1	1	0	1	1	1	0	0	1	0
**DM (0/1)**	0	0	0	1	0	0	0	0	0	1	0	1	0	0	0
**PAD (0/1)**	1	1	1	1	1	0	1	1	1	1	0	1	0	1	0
**CHD (0/1)**	0	1	0	1	0	0	0	0	0	0	0	1	0	1	0
**CKD (0/1)**	1	0	0	0	1	1	1	1	0	0	1	0	0	0	1
**Atorvastatin (mg)**	40	40	0	20	40	0	0	40	20	0	20	20	20	0	0
**CA9 (mRNA) (0/1)**	1	1	1	1	1	1	0	1	1	1	1	1	1	1	1
**CA IX (protein) (0/1)**	1	1	1	1	1	1	0	0	1	1	1	0	1	1	1
**HIF-1** **α (protein) (0/1)**	0	0	1	0	0	1	0	0	0	0	1	0	0	0	0
**s-CA IX (pg/mL)**	53.0	4.5	61.1	52.2	82.6	513.5	3.4	219.5	49.2	127.5	24.0	36.3	30.3	22.8	103.0

AAA—abdominal aortic aneurysm, AHT—arterial hypertension, BMI—body mass index, CKD—chronic kidney disease, CHD—coronary heart disease, F—female, FF—fusiform AAA, M—male, PAD—peripheral artery disease, S—saccular AAA, 0—absent, 1—present.

**Table 4:** In silico analysis of CA9 and aneurysm-associated genes.

		Dataset/Correlation Coefficient			
Gene	Encoded Protein	GSE57691	GSE7084	GSE47472	GSE98278
ACTA2	Actin Alpha 2, Smooth Muscle	0.150747	0.665397	0.815893	−0.119043
CDH5	VE Cadherin	−0.331481	0.651761	0.733326	0.053510
COL1A1	Collagen Type I Alpha 1 Chain	0.247549	0.570638	0.756304	−0.179151
COL3A1	Collagen Type III Alpha 1 Chain	−0.292330	0.657554	0.827058	−0.129495
CTSB	Cathepsin B	−0.022477	−0.432026	0.763813	−0.064998
CTSD	Cathepsin D	−0.048503	−0.157067	0.784142	−0.015472
CTSK	Cathepsin K	−0.360297	0.187544	0.780399	0.091335
CTSL1	Cathepsin L1	0.238726	0.367675	0.786655	0.157626
CTSL2	Cathepsin L2	0.702837	−0.638019	−0.373576	0.279559
CTSS	Cathepsin S	−0.485126	−0.592616	0.755907	0.107137
FOXO4	Forkhead Box O4	0.009577	0.588396	0.769559	−0.076710
MMP8	Matrix Metalloproteinase 8	0.851489	−0.660673	0.683267	0.118283
MMP9	Matrix Metalloproteinase 9	−0.054047	−0.864212	−0.242989	0.230107
MYOCD	Myocardin	0.767331	0.697014	0.714962	−0.073694
SPP1	Secreted Phosphoprotein 1, Osteopontin	0.004500	0.729077	0.765857	0.182772
SRF	Serum Response Factor	−0.365013	0.655447	0.652437	−0.097664
TIMP1	Metallopeptidase Inhibitor 1	0.486787	0.588118	−0.500345	0.414479
TIMP2	Metallopeptidase Inhibitor 2	0.774459	−0.111466	0.690500	0.049871
TIMP3	Metallopeptidase Inhibitor 3	−0.032166	0.496904	0.758487	0.399108
VEGFA	Vascular endothelial Growth Factor A	0.772675	0.717174	0.786801	0.747852
VIM	Vimentin	−0.148257	−0.029205	0.702479	0.132072
XBP1	X-box Binding Protein 1	−0.398026	0.643267	0.743585	0.100872

Color scale: fading red color shades from −1 to −0.2 and increasing intensity of blue color shades from 0.2 to 1.

**Table 5:** Primer sequences.

Gene	Sequence
*CA9*	Sense: 5′-TAAGCAGCTCCACACCCTCT-3′
	Antisense: 5′-AATCACTCGCCCATTCAAAG-3′
ACTA2	Sense: 5′-CCGGGACTAAGACGGGAATC-3′
	Antisense: 5′-TTGTCACACACCAAGGCAGT-3′
β-actin	Sense: 5′-CCAACCGCGAGAAGATGACC-3′
	Antisense: 5′-GATCTTCATGAGGTAGTCAGT-3′
SPP1	Sense: 5′-GCAGACCTGACATCCAGTACC-3′
	Antisense: 5′-TGTGGGTTTCAGCACTCTGG-3′
VEGFA	Sense: 5′-CTTGCTGCTCTACCTCCACCAT-3′
	Antisense: 5′-CACACAGGATGGCTTGAAGATG-3′
CTSD	Sense: 5′-GTACCTGAGCCAGGACACTG-3′
	Antisense: 5′-CGAACTTGGCTGCGATGAAG-3′
TIMP1	Sense: 5′-GCGTGGACATTTATCCTCTAGC-3′
	Antisense: 5′-AAGGTGGTCTGGTTGACTTCTG-3′
TIMP2	Sense: 5′-GGTCAGTGAGAAGGAAGTGGAC-3′
	Antisense: 5′-GTACCTGTGGTTCAGGCTCTTC-3′
TIMP3	Sense: 5′-GCTGTGCAACTTCGTGGAGA-3′
	Antisense: 5′-GTAGCAGGACTTGATCTTGCAGT-3′

## Data Availability

Publicly available datasets were analyzed in this study. This data can be found here: https://www.ncbi.nlm.nih.gov/geo/query/acc.cgi/accession number, accessed on 2 December 2021, GSE57691, GSE7084, GSE47472, GSE98278.

## Abbreviations

AAA—abdominal aortic aneurysm; ACEI—angiotensin-converting enzyme inhibitor; ACTA2—Actin Alpha 2 Smooth Muscle; ADAM17—a disintegrin and metalloprotease 17; AKT- protein kinase B; ARB—angiotensin receptor blocker; BB—beta blocker; BMI—body mass index; CA IX—carbonic anhydrase IX; *CA9*—carbonic anhydrase 9 (gene); CAD—coronary artery disease; CDH5—VE cadherin; CKD—chronic kidney disease, COL1A1—Collagen Type I Alpha 1 Chain; COL3A1—Collagen Type III Alpha 1 Chain, CRP—C-reactive protein; CTSB—cathepsin B; CTSD—cathepsin D; CTSK—cathepsin K; CTSL1—cathepsin L1; CTSL2—cathepsin L2; CTSS—cathepsin S; EF—ejection fraction of left ventricle; ELISA—enzyme-linked immunosorbent assay; FOXO4—Forkhead Box O4; HIF—hypoxia inducible factor; HLP—hyperlipidemia; MMP—matrix metalloproteinase; MYOCD—myocardin; p-AKT—phosphorylated AKT; PI3K—phosphoinositide-3-kinase; SPP1—Secreted Phosphoprotein 1, Osteopontin; SRF—serum response factor; TIMP—tissue inhibitor of matrix metalloproteinase; VEGFA—vascular endothelial growth factor A; VIM—vimentin; VSMC—vascular smooth muscle cell; XBP1—X-box binding protein 1.
